# Diisocyanate-induced asthma in Switzerland: long-term course and patients’ self-assessment after a 12-year follow-up

**DOI:** 10.1186/1745-6673-9-21

**Published:** 2014-05-14

**Authors:** Martin Rüegger, Doreen Droste, Markus Hofmann, Marcel Jost, David Miedinger

**Affiliations:** 1 Department of Occupational Medicine, Swiss National Insurance Fund Suva, Fluhmattstr. 1, CH 6002 Lucerne, Switzerland

**Keywords:** Occupational asthma, Diisocyanate asthma, Long-term follow-up, Self-assessment

## Abstract

**Background:**

Isocyanates are among the most common causes of occupational asthma (OA) in Switzerland. Patients with OA have been shown to have unfavourable medical, socioeconomic and psychological outcomes. We investigated long-term asthma and the socio-economic outcomes of diisocyanate-induced asthma (DIA) in Switzerland.

**Patients and methods:**

We conducted an observational study on 49 patients with DIA and followed 35 of these patients over a mean exposure-free interval of 12 ± 0.5 (range 11.0-13.0) years. At the initial and follow-up examinations, we recorded data on respiratory symptoms and asthma medication; measured the lung function; and tested for bronchial hyperreactivity. We allowed the patients to assess their state of health and overall satisfaction using a visual analogue scale (VAS) at these visits.

**Results:**

The 35 patients whom we could follow had a median symptomatic exposure time of 12 months, interquartile range (IQR) 26 months and a median overall exposure time of 51 (IQR 104) months. Their subjective symptoms (p < 0.001) and the use of asthma medication (p = 0.002), particularly the use of inhaled corticosteroids (p < 0.001), decreased by nearly 50%. At the same time, the self-assessment of the patients’ state of health and overall satisfaction increased considerably according to both symptomatology and income. In contrast, slight reductions in terms of FVC% predicted from 102% to 96% (p = 0.04), of FEV1% predicted from 91% to 87% (p = 0.06) and of the FEV1/FVC ratio of 3%; (p = 0.01) were observed while NSBHR positivity did not change significantly. In univariate as well as multivariate logistic analyses we showed significant associations between age, duration of exposure and FEV1/FVC ratio with persistent asthma symptoms and NSBHR.

**Conclusions:**

We found that the patients’ symptoms, the extent of their therapy and the decrease in their lung volumes during the follow-up period were similar to the findings in the literature. The same hold true for some prognostic factors, whereas the patients’ self-assessment of their state of health and overall satisfaction improved considerably.

## Background

For more than two decades diisocyanates, a group of highly reactive and widely used chemicals, were one of the most common causes of occupational asthma in industrialised countries [1] though the incidence of diisocyanate induced occupational asthma (DIA) seems to have decreased over recent years [2-4].

Follow-up studies published during the last 20 years have shown a poor outcome for DIA even in the absence of additional exposure to the offending agent. Approximately 50-82% of persons who suffer from DIA do not recover completely and need continuous anti-asthmatic therapy [5-8]. According to the literature, recovery depends on a series of prognostic factors, such as the lung volume at the beginning of exposure [9], the time of diagnosis [10,11], the degree of bronchial hyperreactivity [9], the length of exposure time [12,13], the duration of symptomatic exposure [11,14,15] and possibly the duration of follow-up [16,17].

Until the early 1990s, there were only clinical follow-up studies on DIA that explored short time periods [7,8,12,13,18]. Therefore, we decided to do a long-term study with our own DIA patients beginning in 1993 and covering a time period of at least 10 years. Subsequently, in 2000 and in 2003, two carefully conducted studies on the outcome of patients with DIA over a 12-year follow-up were published [19,20].

The aim of our study was to investigate the long-term outcome of DIA in a non-preselected group of patients; we focussed on asthma symptoms, the need for treatment, changes in lung function and on nonspecific bronchial hyperreactivity (NSBHR). Further we wanted to evaluate the factors that were associated with persistent asthma symptoms and NSBHR [16] as well as the individual state of health and overall satisfaction at follow-up.

## Materials and methods

All patients with suspected DIA reported to Suva (Swiss Accident Insurance Fund) between 1993 and 1995 were eligible for the study. Patients were included if the combination of their history and their spirometric findings, peak expiratory flow (PEF) recordings or inhalation challenge test results confirmed the diagnosis [21]. All of the patients gave their informed consent to participate in the study.

The patients who were admitted to our study had been exposed to one or more of the following diisocyanates: toluene diisocyanate (TDI), diphenylmethane diisocyanate (MDI), hexamethylene diisocyanate (HDI), naphthylene diisocyanate (NDI) or isophorone diisocyanate (IPDI) or one of their reactive prepolymers either continuously or intermittently.

We performed an uncontrolled observational study over 12 years. For each subject, a detailed clinical and occupational history was recorded and lung function and bronchoprovocation tests were performed between 1993 and 1995, i.e., at the time of diagnosis (T0), and again in 2006, at the time of re-evaluation (T1). The participants’ longitudinal results are presented with data available at T0 and T1.

The data were recorded by one of Suva’s occupational physicians at three different locations (Lausanne, Lucerne and Winterthur) during 1993–1995 (T0) and again in 2006 (T1) by DD as part of her doctoral thesis except two patients who were interviewed and examined by MH, and two others by their family doctors because of the patients’ limited mobility. To ensure uniformity of questioning a checklist was used that had been established before. Most of the data have been collected routinely when evaluating notified cases in order to determine causality of occupational asthma (OA).

The subject’s history included upper airway symptoms, such as nasal flow and itching, and lower airway symptoms, such as irritation, cough, wheezing, dyspnoea, chest tightness, and their temporal relationship to work. Additionally, information on the use of asthma medications, smoking habits, isocyanate exposure and current workplace as well as socioeconomic aspects were gathered.

In 2006, the subjects were asked to report their state of health as well as their overall satisfaction using two identical 10 cm neutral VASs. These measures reflect subjective factors in a global manner but correlate highly with several standard questionnaires and are accurate and useful for assessing symptoms, such as pain, anxiety and psychological distress [22]. Patients had to enter one mark retrospectively for the time T0 and another mark for the current time, T1. The second VAS mark could not be set until the first one was covered. The left end of the scale (0 cm) signified the worst rating conceivable, whereas the right end (10 cm) indicated the optimal rating.

Between 1993 and 1995, all patients had a walk-through visit by an occupational physician to confirm exposure to isocyanates and to exclude relevant co-exposure to other sensitizers or irritants that might have caused the reported symptoms (results not reported).

All of the patients underwent routine clinical examinations as well as standard lung function tests at one of Suva’s three occupational medical centres. The tests conducted at time T0 and T1 included forced vital capacity (FVC), forced expiratory volume in 1 second (FEV1) and the FEV1/FVC ratio, which in most cases were measured by whole-body plethysmography (Jaeger MasterLab Germany, Vmax Autobox, VIASYS Healthcare Inc., SensorMedics, California, USA). The values published by the European Respiratory Society were used as reference values [23]. A significant obstructive ventilatory impairment was assumed to be present when the FEV1/FVC ratio was <0.7 [24].

To test NSBHR with acetyl-β-methylcholine chloride inhalation, Mefar dosimeters (Mefar MB3, Brescia, Italy) were used. The results were expressed as the cumulative doses required to provoke an FEV1 reduction of 20% (PD_20_) graded according to the ATS scheme which was slightly modified [25]. If the FEV1 was <70% of the predicted value, the test was not performed, and the reversibility was checked by inhaling two puffs of salbutamol (200 μg DA). Generally we defined NSBHR in the participants as having a fall in FEV1 of >20% by inhaling ≤2 mg methacholine or having an increase in FEV1 of >10% after inhalation of salbutamol.

To confirm respiratory tract sensitisation to diisocyanates at T0, 31 of the 49 patients underwent a specific-inhalation challenge in the Thurgau-Schaffhausen Alpine Clinic in Davos according to a standardised protocol with the relevant diisocyanates or isocyanate-containing products. Tests were positive if an FEV1 decrease of at least 20% could be demonstrated and if the dose response curve showed a typically shaped immediate, late or dual reaction [26].

To compare the results between the beginning and the end of the follow-up examinations, only complete data sets were accounted for; i.e., those of dropouts were discarded. In order to check for prognostic factors we also stratified participants according to their FEV1% predicted, FEV1/FVC ratio at T0 and a change in FEV1% predicted between T0 and T1.

Continuous variables are reported as a mean ± standard deviation (SD) or median plus interquartile range (IQR) when non-normally distributed. Statistical tests applied were the Kolmogorov-Smirnov, the McNemar’s-, paired t-, Sign-, Wilcoxons-Mann–Whitney- and the chi-squared test. After performing univariate analyses, a logistic regression analysis with staggered inclusion of co-variates was performed to evaluate the effects of different variables on dependent variables such as “persistent asthma symptoms” and “persistent bronchial hyperresponsiveness at follow-up”. In the “a priori” models we selected some variables (age at diagnosis, total exposure time, exposure time with symptoms) that were already shown to be associated with asthma outcome [16]. In the “a posteriori” models we included co-variates in whom univariate analyses showed significant or borderline associations with the dependent variables. Statistical analyses were performed by applying the PSPP software package [27]. To calculate mean values ± SD, median values and IQRs, Microsoft Excel for Mac Version 14.3.9 software was used. A p < 0.05 was considered to be statistically significant.

## Results

Forty-nine patients (47 males, 2 females; mean age, 38 ± 13 years) were considered for inclusion into the study. These patients represented all individuals who claimed compensation between 1993 and 1995 and for whom the diagnosis of DIA could be established. Most of the study participants were car spray painters, industrial spray painters, furniture spray painters, carpenters and cabinet makers (49%), followed by polyurethane foam casters and moulders (37%) and other craftsmen (14%).

**Figure 1:**
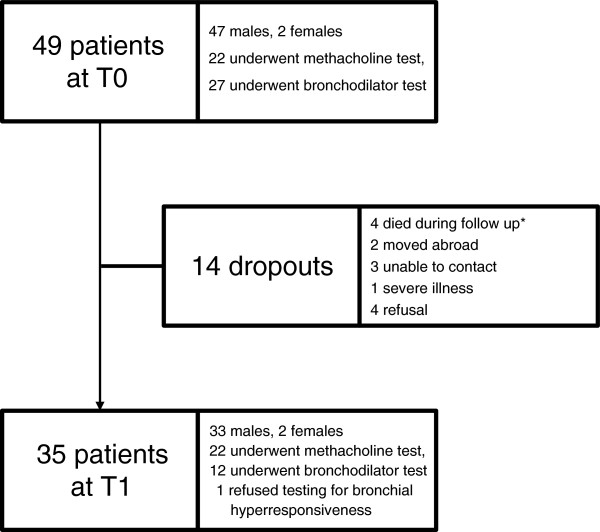
**Flow-chart of recruited patients.** Legend: T0 = time of diagnosis, T1 = time of re-evaluation. *Reasons for death (coronary heart disease (n = 1), aspiration pneumonia (n = 1), unknown (n = 2)).

We observed that 86% of the subjects were no longer being exposed to isocyanates when the diagnosis of OA was made (T0). They had ended their exposure on average one month before the investigation (range, 30 months before to 7 months thereafter) because Suva declared 44 of them unsuitable for further isocyanate-related occupational activities (declaration of “unsuitability” [DOU]) [28]. Five patients could not be declared unsuitable because three of them were self-employed persons, one received an invalidity pension for a non-occupational disease and one was affected from DIA very early during his apprenticeship so that a DOU was not indicated. In practice, the DOU resulted in nearly all of the patients changing their workplace or their employer. The median overall isocyanate exposure time of the whole cohort was 57 months (IQR 108 months), and the median symptomatic exposure time was 16 months (IQR 30 months).After a mean interval of 12 years (range, 11.0–13.0 years), 35 of the 49 patients could be contacted and agreed to be re-examined, corresponding to a re-examination rate of 71.4%. Figure 1 summarizes the flow of patients and the reasons for non-follow up. Twenty-two subjects (63%) were still working. One of them remained at the original workplace as a car spray painter but had switched to non-isocyanate-containing varnishes, 4 (11%) had relocated to completely isocyanate-free workplaces within the same company, and 17 (49%) had changed their job as well as their employer to avoid further isocyanate exposure. Thirteen subjects were no longer occupationally active, 7 due to premature or regular retirement; 3 were on social welfare, 2 were unemployed and one was on long-term sick leave unrelated to DIA.

The characteristics of the 35 participating subjects and the 14 dropouts are shown in Table 1. Except for the lower PD_20_ at T0 for those that were not re-examined later, the other variables did not significantly differ from those of the individuals that were re-investigated for this study.

**Table 1:** Baseline characteristics of participants and dropouts at T0 (n = 49)

	**Participants (n = 35)**	**Dropouts (n = 14)**	**p-value**
Age at diagnosis	39 ± 13	36 ± 13	0.53
Sex (males)	33 (94%)	14 (100%)	1.0
FVC% predicted	102 ± 17	97 ± 13	0.37
FEV1% predicted	91 ± 16	90 ± 16	0.89
FEV1/FVC	75 ± 9	74 ± 10	0.50
PD_20_ (mg)	0.95 (1.7)	0.30 (0.5)	0.03
NSBHR positivity	23 (68%)	11 (92%)	0.13
Total exposure time (months)	51 (104)	74 (89)	0.77
Time with symptoms (months)	12 (26)	24 (31)	0.38
Smokers	10 (29%)	6 (43%)	0.5
Pack-years*	25 (24)	13 (14)	0.16
Upper airway symptoms	28 (80%)	11 (79)	1.0
Asthma symptoms	34 (97%)	14 (100)	1.0
Asthma medication	31 (91%)	8 (89)	1.0

Legend: FVC = forced expiratory vital capacity. FEV1 = forced expiratory volume in 1 second. NSBHR = nonspecific bronchial hyperreactivity. PD20 = provocative dose of methacholine producing a fall in FEV1 of 20%. *Pack-years for smokers and ex-smokers added. Data is presented as mean ± SD, median and interquartile range (IQR) or number (%).

Table 2 represents the changes in symptoms, asthma therapy, health status and overall satisfaction for those participants for whom follow-up data was available. It shows a significant decline in the frequency of symptoms, use of asthma medication - especially inhaled corticosteroids - and an increase in subjective health status and overall satisfaction.

**Table 2:** Change in symptoms, therapy and visual analogue scale (VAS) ratings between T0 and T1 (n = 35)

	**T0**	**T1**	**p-value**
Upper airway symptoms	28 (80%)	9 (26%)	<0.001
Asthma symptoms	34 (97%)	20 (57%)	<0.001
Use of asthma medication*	31 (91%)	19 (56%)	0.002
Short acting beta2 agonists	19 (56%)	13 (38%)	0.18
Long acting beta2 agonists	10 (29%)	10 (29%)	1.0
Inhalable corticosteroids	26 (76%)	10 (29%)	<0.001
Smokers	10 (29 %)	14 (40%)	0.05
VAS (cm) health status	1.2 (2.9)	8.0 (4.0)	<0.001
VAS (cm) overall satisfaction	2.1 (6.1)	8.3 (3.4)	<0.001

Legend: Upper airway symptoms (conjunctivitis, rhinitis, hoarseness, pharyngeal irritant sensations); asthma symptoms (irritation, wheezing, chronic cough, chest tightness and dyspnoea); VAS = visual analogue scale. Data is presented as mean ± SD, median and interquartile range (IQR) or number (%).

*n = 34.

Table 3 summarizes the results of pulmonary function tests for the 35 subjects who were re-examined for this study. Lung function values were slightly lower at T1 compared to T0 reaching the level of significance only for FEV% predicted and for the FEV1/FVC ratio. 34 of 35 participants were tested for NSBHR either by methacholine challenge or by bronchodilatation with salbutamol. While 23 subjects (68%) were positive at the time of diagnosis their number decreased to 17 (50%) at T1, a result that did not reach statistical significance (p = 0.22). When focussing on the 26 participants whose tests were done by the same method on both occasions (22 by methacholine and 4 by bronchodilatation) the number of positive results again dropped from 18 to 15 (14 methacholine and 4 bronchodilatation tests at T0; 14 methacholine and 1 bronchodilatation test at T1) a decline that again did not reach statistical significance. The same was true for those 22 patients who underwent methacholine challenge tests (median of PD_20_ 0.95 mg (IQR 1.68 mg) at T0 versus 0.70 mg (IQR 1.66 mg; p = 0.81) at T1.

**Table 3:** Results of lung function and hyperreactivity testing at T0 and T1

	**T0**	**T1**	**p-value**
FVC% predicted	102 ± 17	96 ± 17	0.04
FEV1% predicted	91 ± 16	87 ± 18	0.06
FEV1/FVC	76 ± 9	73 ± 8	0.01
PD_20_ (mg)	0.95 (1.7)	0.70 (1.7)	0.81

Legend: FVC = forced vital capacity. FEV1 = forced expiratory volume in one second. PD20 = provocative dose of methacholine causing a 20% fall in FEV1. NSBHR = nonspecific bronchial hyperreactivity.

Data is presented as mean ± SD or median and interquartile range (IQR).

Tables 4, 5 and 6 show baseline characteristics according to lung function at T0 and lung function loss during follow-up. Age, FEV1/FVC, smoking variables and exposure data in those individuals with FEV1 < 80% predicted at T0 were not different from those with better lung function. The same was true for those with ≥15% loss of FEV1% predicted between T0 and T1. Individuals with a FEV1/FVC ratio <0.7 at T0 had a lower FEV1% predicted and a higher tobacco consumption than those with higher FEV1/FVC ratio.

**Table 4:** Baseline characteristics of patients according to their FEV1 at T0

	**FEV1 ≥ 80% pred. (n = 28)**	**FEV1 < 80% pred. (n = 7)**	**p-value**
Age	38 ± 13	42 ± 10	0.44
FEV1/FVC	77 ± 8	72 ± 11	0.20
Current smoking	8 (29%)	2 (29%)	1.0
Pack years*	7 (15)	20 (13)	0.37
Total exposure time (months)	51 (99)	53 (129)	0.68
Symptomatic exposure time (months)	13 (34)	8 (14)	0.22

Legend: FVC = forced expiratory vital capacity. FEV1 = forced expiratory volume in 1 second. *Pack-years for smokers and ex-smokers added.

Data is presented as mean ± SD, median and interquartile range (IQR) or number (%).

**Table 5:** Baseline characteristics of patients according to their FEV1/FVC ratio at T0

	**FEV1/FVC ≥ 0.7 (n = 25)**	**FEV1/FVC < 0.7 (n = 10)**	**p-value**
Age (years)	37 ± 14	42 ± 9	0.34
FEV1% predicted	95 ± 13	83 ± 20	<0.001
Current smoking	7 (28%)	3 (30%)	1.00
Pack years*	5 (12)	20 (20)	<0.001
Total exposure time (months)	44 (80)	145 (143)	0.16
Symptomatic exposure time (months)	11 (15)	19 (33)	0.31

Legend: FVC = forced expiratory vital capacity. FEV1 = forced expiratory volume in 1 second. *Pack-years for smokers and ex-smokers added.

Data is presented as mean ± SD, median and interquartile range (IQR) or number (%).

**Table 6:** Baseline characteristics of patients according to their FEV1 loss between T0 and T1

	**FEV1 loss < 15% (n = 28)**	**FEV1 loss ≥15% (n = 7)**	**p-value**
Age	37 ± 13	45 ± 10	0.11
FEV1% pred.at T0	90 ± 17	98 ± 13	0.25
FEV1/FVC at T0	77 ± 9	72 ± 7	0.25
Current smoking	8 (29%)	2 (29%)	1.0
Pack years*	11 (17)	11 (17)	0.42
Total exposure time (months)	46 (91)	62 (169)	0.32
Symptomatic exposure time (months)	11 (19)	19 (49)	0.40

Legend: FVC = forced expiratory vital capacity. FEV1 = forced expiratory volume in 1 second. *Pack-years for smokers and ex-smokers added.

Data is presented as mean ± SD, median and interquartile range (IQR) or number (%).

The results of the multivariate logistic regression analysis can be seen in Table 7. In the “a priori” models age was associated with persistent asthma symptoms at follow-up (p = 0.02) and persistent NSBHR was associated with total exposure time (p = 0.04). In the predictive “a posteriori” model the degree of airflow limitation at diagnosis was associated with persistent asthma symptoms at T1 (p = 0.05). Asthma medication (p < 0.001) and age (p = 0.06) at T1 were associated with asthma symptoms both at follow up.

**Table 7:** Multivariate logistic regression analysis of predictors for persistent asthma at follow-up

	**“A priori”**		**“A posteriori”**	
			**Predictive**	**Descriptive**
	**Model 1**	**Model 2**	**Model 3**	**Model 4**
**Dependent variables**	**Persistent asthma symptoms at follow-up**	**Persistent NSBHR at follow-up**	**Persistent asthma symptoms at follow-up**	**Persistent asthma symptoms at follow-up**
**Co-variates**				
Age at T0	0.08 (p = 0.02)	0.03 (p = 0.35)	0.04 (p = 0.27)	
Age at T1				0.09 (p = 0.06)
Total exposure time		-0.02 (p = 0.04)		
Symptomatic exposure time	0.01 (p = 0.37)	0.03 (p = 0.10)		
Upper airway symptoms at T0			-1.91 (p = 0.12)	
Upper airway symptoms at T1				1.36 (p = 0.29)
FEV1/FVC at T0			-0.11 (p = 0.05)	
Asthma medication at T1				2.85 (p < 0.001)
Intercept	-2.90	-0.32	9.01	-5.98
R^2^	0.22	0.28	0.39	0.55

Legend: FVC = forced expiratory vital capacity, FEV1 = forced expiratory volume in 1 second. NSBHBR = non specific bronchial hyperreactivity. R^2^ = Nagelkerke R Square. Data are presented as B-coefficient (p-value).

## Discussion

The aim of our 12-year follow-up study was to investigate the long-term outcome of patients with DIA, to check for possible prognostic factors and to compare these results obtained in Switzerland with those reported in the literature, primarily of the two previously cited larger cohorts of patients with DIA [19,20]. We found that at a mean of 12 years after removal from exposure to the offending diisocyanates, the majority of the patients reported fewer respiratory symptoms, decreased asthma medication use and a better self-reported overall health status while lung function values at follow up were slightly lower compared to normal.

At T1, 14 of the initially evaluated 49 patients at T0 could not be retraced (dropout rate 28.6%). The 35 patients who could be followed until T1 represent a small sample size, a fact that weakened the informative value of our results. Piirilä (8% non-responders in their first phase questionnaire survey) and, particularly, Padoan and co-workers (no non-responders) did clearly better in this regard. Nevertheless, the 71.4% who re-participated in our study were within the range of other but shorter follow-up studies [15,29-32]. When analysing our data retrospectively, dropouts were having a more pronounced bronchial hyperreactivity (p = 0.03) at diagnosis compared to participants. With respect to all the other variables such as smoking status, job groups (data not shown), age, the presence of airway symptoms, the level of FVC and FEV1% predicted as well as the FEV1/FVC ratio or the overall exposure time period, we could not find any significant differences between study participants and dropouts. Thus we believe that the participants represent fairly well the whole study group, a fact that might outweigh to some extent its small sample size.

Based on the average number of the Swiss working population of 4 millions during 1993 – 1995 (Swiss Federal Statistical Office) the 49 DIA cases correspond to a yearly incidence rate of 4.1 cases/million which is somewhat higher than in France by ONAP indicating 3.5 cases/million [33] but considerably lower than the 10.8 cases/million reported by the PROPULSE program in Québec, Canada [34]. To note that both these sentinel programs became active during the 1990ies i.e. during the same time period when we were following-up our patients and – more important - that we report on legally compensated cases only unlike those published from Québec and from France.

Removing an asthmatic worker from the offending agent leads to an improvement in asthma severity, a fact that was confirmed by Rachiotis and co-workers [16] when doing a systematic literature review and meta-analysis on the outcome of occupational asthma after cessation of exposure. The authors found a pooled rate of symptomatic recovery of 32% (95% CI 26%–38%). This finding is in agreement with the results of a recent review issued by Baur and co-workers [21], who found that, on average, 33.7% of patients (95% CI 23.6%–45.6%) no longer exhibited symptoms after complete cessation of exposure to the offending agent. These results indicate that usually more than 50% of the subjects remain symptomatic, which is in line with our figures showing that 57% of the patients continued to complain of asthma symptoms at follow up, a finding that is slightly better than that observed in the studies by Piirilä and Padoan [19,20], in which more than two thirds of the patients remained asthmatic.

In our study most of the patients were still on anti-asthmatic medication at follow-up; more than half of them were taking it on a regular basis, whereas 10 were still inhaling corticosteroids. These results are comparable to those of both the Piirilä and the Padoan studies, in which 66% and 60%, respectively, of the patients required some form of anti-asthmatic treatment at the end of their follow-up, approximately half of them again on a regular basis in the Piirilä study [19]. According to an extended literature survey that was conducted by the Agency for Healthcare Research and Quality (AHRQ), the proportion of OA patients who require medication after cessation of exposure ranges from 17% to 100%, depending to a great extent on the characteristics of the cohort and on exposure. Furthermore, the AHRQ data suggest that 4 years after removal of the offending low-molecular-weight agent, approximately half of the patients are still under treatment [35], which is in line with what we have observed.

In contrast to the Piirilä and the Padoan studies, we asked the patients to assess their individual state of health as well as their overall satisfaction using a VAS. To the best of our knowledge, these factors have not been addressed in another comparable long-term DIA study. On a group basis, we observed a statistically significant increase in self-reported health status and overall satisfaction between T0 and T1, results that were comparable to those of the Karvala study, in which the overall quality of life parameter in patients with occupational asthma to moulds was reported to be between 6.5 and 7.4 [36]. When we stratified our VAS ratings according to the presence or the absence of symptoms, we observed that those without asthma symptoms at follow-up were reporting even better satisfaction than those with persistent symptoms (data not shown). Assuming that by far the majority of “normal persons” would not set their VAS mark precisely on 10 but rather somewhat lower, our results for the group without symptoms at follow-up do not likely differ from what could be expected from healthy people.

We admit that VAS results have to be interpreted with caution. First of all one can argue that patients who could not be followed up – dropouts - would have been more seriously diseased judging their quality of life significantly lower than the participants thus biasing the observed results in a too optimistic direction. However we think that this hypothesis is not very probable, as – with the exception of PD_20_ – there were no significantly different baseline characteristics between participants and dropouts (Table 1). Secondly there is no doubt that any self-assessment, especially if done retrospectively, is subjective and can thus be biased. However we had no indication for subjects to under or over report their state of health at T0 or T1 except that – in theory - some over-reporting could have been expected because the interviewers were physicians who were employed by Suva the compensating body. Such a tendency could have biased the VAS markings towards a lower level at T0 as well as at T1 thus not decisively change the absolute amount of improvement we observed.

Complete cessation of exposure to the offending agent improves symptoms as well as lung function [37] but is associated with worse socioeconomic outcomes [15,35,38], thereby increasing anxiety and depressive symptoms [31,39-41] that, in turn, have a negative impact on asthma outcome. When examining the data on socioeconomic factors at T1, we found that those 13 patients who were not working lost on average 19.2% of their income, 7 of them after retiring regularly or prematurely, incurring an income reduction either way. Those who were still economically active (22 of 35) had a salary increase of 13.7% (after retraining) or a slight loss of 6% (without retraining) [42]. To look at the impact of both symptoms and income on overall satisfaction at the end of the follow-up, we found that persisting asthmatic symptoms entailed a clearly lower VAS ranking than the loss of income. It is obvious that the latter does not reflect all aspects of possible socioeconomic problems. Nevertheless, we think that once financial problems have been managed and an alternative job is found, to the patients’ satisfaction, psychological distress will be alleviated, resulting in better tolerability of the remaining symptoms, as observed by Piirilä and Keskinen [19,43].

Lung function in the participants was lower than those reported in the Piirilä and the Padoan studies (99.6% and 98.8% predicted respectively) [19,20]. According to the literature, the lungs are not completely developed by late adolescence, with lung function values not plateauing until early in the third decade of life [44]. Two of our 35 subjects were only 17 and 18 years old at diagnosis, a fact that could explain the slight reduction in the average lung volumes at the beginning of the study but not an isolated decrease in FEV1.

The decreases in FVC% predicted as well as in the FEV1/FVC ratio between T0 and T1 were small but significant, whereas the FEV1% predicted changed only insignificantly even though the number of patients with overt airflow limitation remained numerically constant (n = 10). These results are again consistent with those of Piirilä, Padoan and others, indicating slight but overt lung function deterioration during follow-up.

When specifically looking at the observed small FEV1/FVC decrease, which is based on a fixed lower limit of 0.7 instead of using calculated limits of normal, as has been recommended earlier [45,46], we believe that this decrease might be attributed, at least in part, to the physiological loss of elastic recoil by ageing. This argument holds true only if one assumes that any substantial recovery of the lung volume of our subjects occurred during the approximately one-month time period between the cessation of exposure and the examination at T0, outweighing the observed volume decrease. In any case it is questionable whether the observed small lung function decreases are clinically relevant. Another possible explanation for the decrease in lung volumes might be persisting diisocyanate exposure. However, this can be largely ruled out because 86% of the subjects had been removed from any further contact by the time of T0, although 9 persons reported having had sporadic and unintended diisocyanate exposures during the follow-up period.

Overall there was a trend for a decrease in the number of individuals with NSBHR having either a positive challenge test to methacholine or a significant reversibility after inhalation of a short acting bronchodilatator. However when considering PD_20_ in individuals who underwent methacholine testing at the beginning and at the end of the follow up period, no significant change could be observed. It is well known that the duration of symptomatic exposure and the level of NSBHR are risk factors for persistent NSBHR and that some individuals keep their NSBHR despite complete removal from the exposing allergen in the workplace [16,21,47]. We believe that two factors might explain our result: Firstly our NSBHR test pairs are incomplete, potentially biasing the result in favour of those subjects doing worse; and secondly we observed an overall increase of active smokers during follow-up (Table 2), which by itself might be a risk factor for NSBHR [48].

When analysing our participants according to their lung function at T0 or its change during follow up (Tables 4, 5 and 6) we did not find any significant differences concerning age and exposure parameters between affected and non affected individuals, FEV1 and tobacco consumption for the patients with a FEV1/FVC ratio <0.7 (Table 5) excluded, though some of the criteria we looked at are known prognostic factors of OA [9,16,21].

The presence of airflow limitation is a known prognostic factor for asthma, COPD and other (respiratory) diseases. When we compared the patients having an FEV1/FVC ratio <0.7 with those who were non-obstructive at T0 (Table 5) we found the former to have started with a significantly lower FEV1% predicted and to have had a significantly higher tobacco consumption while the other parameters did not differ between the two groups (Table 5) [16,21]. Again these results are very well comparable with what has been published in the literature except for tobacco consumption that is not an accepted risk factor for DIA [6,9]. Nevertheless we assume that in some of our patients airflow limitation at T0 was due to both isocyanate as well as tobacco exposure, the latter playing an explicit role as aggravating factor as it is known that smoking adversely affects asthma outcome due to altered airway inflammation and corticosteroid insensitivity contributing therefore to a high rate of symptom persistence and lung function loss at follow-up [49]. Further it is possible that some of our subjects started their isocyanate exposure with a pre-existing airflow limitation, a hypothesis we cannot prove because we do not dispose of any medical data for the time when they started their isocyanate exposure.

When we further stratified our cohort according to the FEV1 loss that was observed between T0 and T1 (Table 6) we largely obtained the same results: The “marked decliners” (FEV1 loss ≥15% predicted) tended to be older, to have a lower FEV1% predicted at T0 and a longer total diisocyanate as well as a symptomatic exposure time.

Finally we tried to identify factors that were associated with the long-term outcome such as persistent asthmatic symptoms and NSBHR by performing a multivariate logistic regression analysis. Several studies did report that duration of (symptomatic) exposure and age at diagnosis are associated with adverse outcomes of occupational and non-occupational asthma [9,16,21]. This is what we could confirm in the “a priori model” as age and exposure time were associated with DIA outcomes. In the “a posteriori model” the FEV1/FVC ratio was the factor that best predicted persistent asthma symptoms at follow-up. Finally age and asthma medication at follow-up were the co-variates that were associated with persistent asthma symptoms at follow-up. To note that age might be linked to the length of exposure, two variables that have not been clearly disentangled until now [9]. However in our sample age and total exposure time did not correlate at all (Pearson Correlation Coefficient = 0.11, p = 0.47) while total exposure time showed only a moderate correlation with symptomatic exposure time (Pearson Correlation Coefficient = 0.53, p < 0.001). Rachiotis and co-workers found - based on 2 studies only – older patients to be more likely to completely recover from NSBHR while 5 out of 6 other cited studies reported shorter durations of exposure to be associated with a better outcome in terms of physiological recovery respectively NSBHR. Rachiotis’ result is supported – at least in tendency - by Baur and colleagues [21] who pointed to the fact that the data from the literature they based their report upon were mostly not significant. To conclude there is no firmly established association between NSBHR and independent variables such as age and duration of exposure while NSBHR positivity at the time of diagnosis is a negative prognostic factor [9,21].

The finding of an association between the FEV1/FVC ratio at T0 and the persistence of asthma symptoms at T1 is supported by the review of Maestrelli and co-workers who stated that impaired lung function at diagnosis had a negative role on the outcome of OA though they report only isolated data on FEV1 and FVC but not on their ratio.

In retrospect, we recognised that medical examinations dating back to the start of exposure would have been very helpful enabling us to look for individuals at risk when further exposed to noxious substances at their workplaces. As part of preventive measures, we would recommend reasonable medical pre-employment or at least surveillance programs to be introduced not only for all isocyanate-exposed workers but for all persons at risk to develop occupational asthma [1,21,50,51].

## Conclusion

In conclusion, we found that more than 50% of our 35 subjects suffering from DIA reported to remain symptomatic, had worse lung function and were in need of therapy for asthma after an exposure-free follow-up period of 12 years. In contrast, the patients’ self-assessment of their health and overall satisfaction increased considerably during the observation period, which is primarily a consequence of becoming symptom free and having satisfactorily resolved their socioeconomic problems. Persistence of asthma symptoms and NSBHR was associated with age, exposure time and lung function at the time of diagnosis (T0). Our results suggest that surveillance programs as well as information on occupational risks for asthma and early symptoms recognition should be offered to the workforce thereby not excluding older and experienced workers as they are still at risk for DIA the latter being characterized by worse outcomes. Finally workers and especially patients with diagnosed occupational asthma should receive counselling on smoking cessation as continuous smoking may adversely affect asthma outcomes.

## Abbreviations

DIA: Diisocyanate asthma; NSBHR: Non-specific bronchial hyperreactivity; DOU: Declaration of unfitness; OA: Occupational asthma; PEF: Peak expiratory flow; VAS: Visual analogue scale.

## Competing interests

The authors declare that they have no competing interests.

## Authors’ contributions

MR was involved in the initial planning of the study. He also drafted and revised the manuscript. DD performed all but four of the follow-up examinations. She also wrote a doctoral thesis evaluating additional data that were not included in the present paper. MH conducted two of the remaining follow-up examinations. MJ was the initiator of the study and the former chief occupational physician and head of Suvas’ occupational medical department. DM critically revised the study results and contributed substantial medical as well as statistical input. All of the co-authors read and approved the final manuscript.
